# Automated, Laboratory-based System Using the Internet for Disease Outbreak Detection, the Netherlands

**DOI:** 10.3201/eid0909.020450

**Published:** 2003-09

**Authors:** Marc-Alain Widdowson, Arnold Bosman, Edward van Straten, Mark Tinga, Sandra Chaves, Liesbeth van Eerden, Wilfred van Pelt

**Affiliations:** * European Programme for Intervention Epidemiology and Training, Bilthoven, the Netherlands; † National Institute for Public Health and the Environment (RIVM), Bilthoven, the Netherlands

**Keywords:** Disease outbreaks, algorithms, Internet, laboratories, data collection

## Abstract

Rapid detection of outbreaks is recognized as crucial for effective control measures and has particular relevance with the recently increased concern about bioterrorism. Automated analysis of electronically collected laboratory data can result in rapid detection of widespread outbreaks or outbreaks of pathogens with common signs and symptoms. In the Netherlands, an automated outbreak detection system for all types of pathogens has been developed within an existing electronic laboratory-based surveillance system called ISIS. Features include the use of a flexible algorithm for daily analysis of data and presentation of signals on the Internet for interpretation by health professionals. By 2006, the outbreak detection system will analyze laboratory-reported data on all pathogens and will cover 35% of the Dutch population.

Rapid detection of outbreaks on a time scale compatible with disease incubation periods is recognized as crucial to maximize the effect of control measures. Most outbreaks are rapidly detected and controlled locally. However, outbreaks involving cases over a wider area or in several local health jurisdictions may have only few local cases and thus be easily missed, especially if the outbreak has a slowly rising number of cases. Outbreaks of certain pathogens with common signs and symptoms (e.g., gastroenteric disease) can also be missed. The role of national laboratory data in detecting such outbreaks has been increasingly recognized in the last few years as modern typing techniques give more precision on the pathogen type and subtype, routinely unearthing outbreaks by linking cases either locally, nationally, or internationally (*1*–*4*) that otherwise would probably not be detected. In addition, surveillance of a wide range of pathogens is essential in identifying emerging disease threats (*5*,*6*). The increasingly perceived threat of bioterrorism recently has made more urgent the need for rapid detection of increases in laboratory diagnoses of common and uncommon pathogens to complement clinician-based reporting systems. Increasing computational power in the last 10 years has resulted in the development of mathematical algorithms to routinely and rapidly detect significant clusters within large amounts of surveillance data (*7*–*12*). Automated electronic laboratory reporting is frequently promoted to improve data quality and timeliness of collection (*13*). More recently, the general availability of the Internet permits feedback to many users, who can have continuous, simultaneous, and even interactive access to information. The Internet allows for immediate communication of signals of possible outbreaks to relevant professionals for interpretation and action.

In the Netherlands, these developments have led to the implementation of automated laboratory-based surveillance system integrated with the Internet in a project named the Infectious Disease Surveillance Information System (ISIS). We describe the development of an automated outbreak detection system within ISIS for all laboratory-reported pathogens in the Netherlands. The system is updated daily with Web-based feedback.

## Overview of National Laboratory Surveillance

In the Netherlands, >90% of the 76 microbiologic laboratories are associated with public hospitals; <10% are private laboratories not associated with hospitals. Other than 10 notifiable infectious diseases, microbiologic laboratories have no legal requirement to provide data for surveillance. Since 1994, ISIS has collected anonymous positive and negative test results on over 350 pathogens directly from voluntarily participating laboratories on a daily basis in a fully automated system that uses electronic data interchange. The raw information is then processed by applying a set of criteria based on the diagnosis of a particular infection. Laboratory results are thus combined into surveillance diagnoses by the removal of results of duplicate testing of the same case by the same or a different microbiologic technique and then classified by the type of infection^1^. Surveillance diagnoses are then presented as feedback on a password-protected Internet site within 24 hours. At present, information on 40 of the 350 pathogens is presented on this site (Table) (available with password at: URL: http://www.isis.rivm.nl). Currently, 11 laboratories located throughout the country are connected to ISIS, covering 16% of the total Dutch population of 16 million. The coverage of each laboratory is calculated from the coverage of each hospital exclusively served by that laboratory, which in turn is calculated by a national organization that calculates the government subsidy to each hospital. One laboratory (the National Institute for Public Health and the Environment [RIVM]) is also the national reference laboratory for *Salmonella*, *Escherichia coli*, and *Mycobacterium tuberculosis*, for which the coverage is much higher. The coverage of the *Salmonella* reference laboratory, for example, is estimated to be 64% of the national population. Since 1996, an algorithm has been used to detect outbreaks in the surveillance data resulting from *Salmonella* (sub)typing (*14*).

**Table:** List of 40 current surveillance diagnoses generated on ISIS with type of threshold^a^

Surveillance diagnosis	Threshold type
Adenovirus infection	H
*Entamoeba histolytica,* intestinal infection	H
*Entamoeba histolytica,* extraintestinal infection	H
*Campylobacter* spp. infection	H
Campylobacter *jejuni* infection	H
*Chlamydia trachomatis* infection	H
Enterovirus infection	H
*E. coli* O157 infection	F (4)
*Giardia lamblia* infection	H
*Neisseria gonorroeae* infection	H
*Haemophilus influenza,* invasive infection	H
Hepatitis A virus infection	H
Hepatitis B virus infection	H
Hepatitis C virus infection	H
*Bordetella parapertussis* infection	H
*Bordetella pertussis* infection	H
Hantavirus infection	F (0)
*Listeria monocytogenes* infection	F0
Malaria *Plasmodium spp* infection	H
Malaria, *Plasmodium ovale* infection	H
Malaria, *P. malaria* infection	H
Malaria, *P. falciparum* infection	H
Malaria, *P. vivax* infection	H
*Neisseria meningitis,* invasive infection	H
Parainfluenza virus infection	H
*Salmonella enterica* Paratyphii group A infection	H
*S.* Paratyphii group B infection	H
*S.* Paratyphii group C infection	H
*S.* Typhi infection	F (3)
Respiratory syncytial virus infection	F (10)
Rhinovirus infection	F (10)
*Salmonella* spp. (nontyphoid) infection	H
*S.* Typhi infection	H
*Shigella* spp*.* infection	F
*Staphylococcus aureus,* invasive infection	H
*Streptococcus* group A, invasive infection	H
*Streptococcus* group B, invasive infection	H
*Streptococcus pneumoniae* infection	H
*Yersinia* spp., non-pestis	H
*Yersinia enterocolitica*	H

^a^ISIS, Infectious Disease Surveillance Information System; H, historical algorithm-defined threshold; F, fixed user-defined threshold (cases/week); F0, zero threshold where one case is signaled.

Apart from ISIS, two other systems collect laboratory data. Fifteen regional public health laboratories provide a weekly report of aggregated data of positive diagnoses for nine bacterial pathogens. These same laboratories and two other laboratories form a network of 17 virologic laboratories that report weekly aggregated numbers of positive diagnoses of 37 virologic pathogens. Four of the 15 public health laboratories contribute data electronically to ISIS.

## Design of the Outbreak Detection System

The overall objective of the system was the automated detection of an unexpected national increase of any one pathogen reported by laboratories in a determined period, for feedback to all interested parties by means of the Internet, followed by interpretation and communication to relevant authorities for decisions on control to be taken. The system thus comprises three components: detection of clusters in time or unusual disease events (e.g., one case of rabies) and signal generation; feedback of the signals on the Internet to relevant professionals; and interpretation of signals on a weekly basis with communication to relevant authorities.

## Cluster Detection and Generation of Signals

### Approach

Our approach was to design a system to detect outbreaks that otherwise would probably be missed altogether and detect more rapidly the outbreaks that would also probably be eventually detected by other means. We designed the system with sensitivity and timeliness as the priority features, especially since small increases in laboratory data often indicate larger communitywide outbreaks. Sensitivity in this context would be defined as the number of relevant outbreaks found from all relevant outbreaks. Clearly, this distinction depends on how “relevant” is defined. All relevant outbreaks, however, should include those outbreaks of public health importance that are missed by conventional means; therefore, the denominator will always be unknown. Thus, absolute sensitivity of the automated system will be impossible to calculate. The system, however, can be designed to maximize sensitivity and detect more outbreaks than other mechanisms such as clinical observation, without resulting in an unmanageable number of signals. The system was also intended to be more timely*,* by detecting the same outbreaks as other mechanisms but more quickly. The specificity of the system was considered less important in the initial phase, since false-positive results could be filtered out when signals were interpreted.

We also decided that the system should be sensitive enough to detect even one case of certain critical infectious diseases (e.g., hantavirus infection) or unusual infections of current interest (e.g., hepatitis E virus infection), which might indicate an outbreak, and to detect expected seasonal increases of diseases caused by selected pathogens (e.g., influenza) as they occur. This design would allow for rapid action to verify the signal and institute case-finding or put in place certain public health measures (e.g., prompting nursing homes to vaccinate residents against influenza).

### Generation of Signals

Signals generated by the system are produced by comparing observed values with a predefined threshold value. Threshold values are calculated from values expected from historical data (for most pathogens) or are fixed, user-defined thresholds, set by epidemiologists for detecting seasonal increases or monitoring critical pathogens.

### Algorithms Using Historical Data

Several algorithm types applied to outbreak detection have been described in the literature, based either on Cumulative Sums (*12*,*15*) linear regression (*7*), or Fourier regression and autocorrelative models such as Box-Jenkins (*8*,*9*). Fourier analysis and autocorrelative methods require model building or the setting of many parameters, processes considered too labor-intensive for a generic algorithm for all type of pathogens. We decided to base the ISIS system on the algorithm currently run each week on *Salmonella* data, which has been successfully detecting outbreaks since 1998 in the Netherlands (*14*,*16*–*20*) but is not automatic and requires an operator to periodically update data. The algorithm is a simple linear regression model, adjusted for seasonality, secular trends, and past outbreaks in a similar manner as described by Farrington et al. (*7*) and requires little parameter resetting or model checking. Briefly, to calculate an expected total value for the current epidemiologic week, a regression line is plotted through the totals in the nine epidemiologic weeks centered on the same epidemiologic week in the previous 5 years. For example, to calculate an expected value for week 20, a regression line is plotted through the values at weeks 16–24 of the previous 5 years.

To maximize sensitivity we decided, after preliminary testing with *Salmonella* data, on two variations of the same algorithm, using two different window periods. The first is a 7-day total calculated daily. This variation is based on an algorithm that calculates expected week totals of a certain pathogen and a threshold value of 2.56 standard deviations from the mean (equivalent to a 99% confidence interval). A 7-day window advances day-by-day as new data enter the system and a new 7-day observed total is calculated daily and compared with the expected value for that epidemiologic week (Monday to Sunday). If the observed total is over the threshold, a signal is generated. The second algorithm variation is a 4-week total calculated daily. Each week, this algorithm calculates an expected total of the previous 4 weeks and a 99% threshold value. A 4-week window advances day-by-day and a new 4-week observed total is compared with the expected total for the four epidemiologic weeks ending with the current week.

Most outbreaks would be detected in a timely manner by the 7-day total system. However, comparison of the two algorithms using *Salmonella* data has shown that small sustained increases >1 month would be missed by a 7-day total system, since the threshold value would not be exceeded in any one 7-day period. Including a 4-week total algorithm in the system produces 10% extra signals of outbreaks with slowly increasing numbers of cases, which otherwise would not be detected.

If the 4-week total is <5, or the 7-day total is <3, no signals are generated, even if above threshold. Though reducing sensitivity, this cutoff greatly reduces the number of signals of sporadic cases of infrequent infections that are of little public health significance. The system uses the date the sample was taken for calculation of observed and expected totals since for any one pathogen a variable delay between date of disease onset and date of reporting of result to ISIS is likely. In the case of an outbreak, the use of date or reporting for surveillance would result in a lower peak number of cases spread over a longer period (a “smeared” epidemiologic curve), reducing the sensitivity of the system. Using date of sampling entails retrospective examination of data to ensure that data reported in 1 week and plotted by date of sampling do not produce a signal in weeks previous to reporting. The “look-back” period (i.e., the period of retrospective examination) has been set at 10 epidemiologic weeks. This window allows enough time for most pathogens to be sampled, tested, and reported. New signals of an excess of cases at time of sampling >10 weeks previous to reporting are unlikely to signify unrecognised outbreaks that can still be investigated and controlled.

### User-Defined or Fixed Threshold.

Algorithms depending on automated evaluation of historical data are often unreliable in detecting seasonal increases in pathogens whose seasonality shifts. A flexible, user-defined, fixed threshold was chosen to detect such increases in selected pathogens. For instance, with present historical data on respiratory syncytial virus, 10 positive laboratory results in any epidemiologic week have always indicated the beginning of the epidemic season. Thus, the threshold for that virus is set at 10 positives in a 7-day period. Some pathogens (e.g., hantavirus) have been defined as zero-tolerance, where one positive result is considered worth a signal. Although such cases are often communicated faster by other means, in some of these situations the system can be considered as a backup.

### Data Used

**Figure 1:**
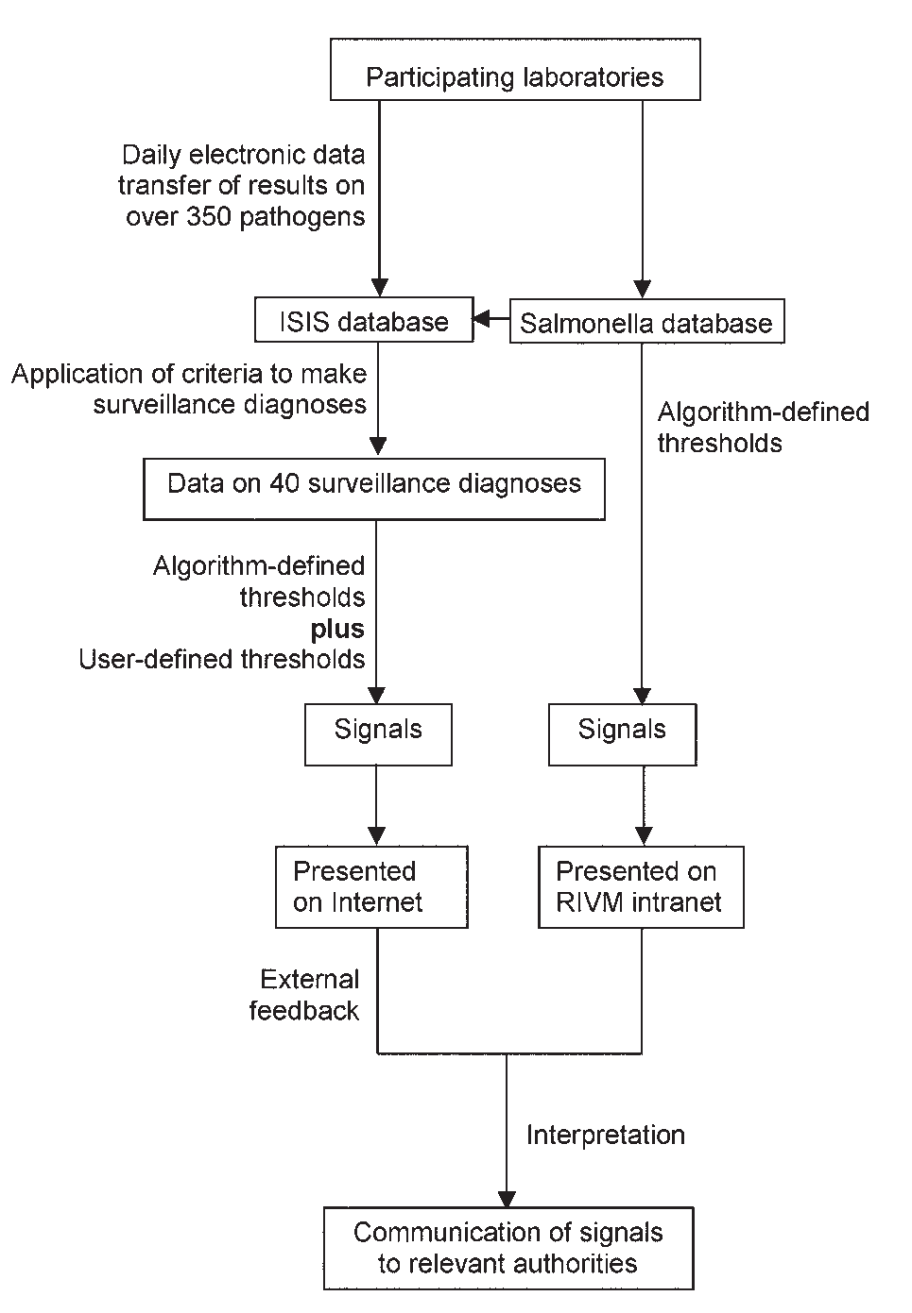
Flow diagram showing flow and processing of laboratory data in the Infectious Disease Surveillance Information System (ISIS) and means by which signals generated by the ISIS database and the *Salmonella* database are created and handled. RIVM, National Institute for Public Health and the Environment, Bilthoven, the Netherlands.

At present, signals are generated from both the *Salmonella* database (data from the national reference laboratory stored in ISIS but not processed into surveillance diagnoses and presented only internally) and the database of 38 surveillance diagnoses (data on pathogens stored in ISIS and processed into surveillance diagnoses for Internet feedback). Signals are generated from the *Salmonella* database with algorithms that use historical data and from the surveillance diagnoses database with both user-defined and algorithm-defined thresholds (Figure 1).

## Internet Feedback of Signals

**Figure 2:**
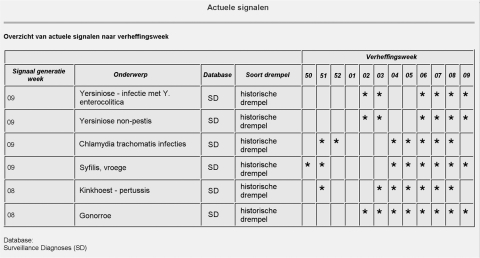
View of Webpage listing surveillance diagnoses (“*onderwerp*”) flagged on week 9 of 2002. The asterisks in the columns labeled “*verheffingsweek*” indicate the week of sampling when the number of a particular surveillance diagnosis exceeded the threshold defined by an historical algorithm (“*historische drempel*”). The surveillance diagnosis for syphilis (“syphilis, *vroege*”) is flagged at the end of 2001 (weeks 51 and 52) and 2002 (weeks 4–9).

Currently, signals from the *Salmonella* database are presented only on an internal RIVM site. Signals are listed and incidence by municipality mapped. The signals generated from surveillance diagnoses, however, are available on the Internet for all local health authorities, Ministry of Public Health staff, and all registered microbiologists to access. The signals are presented first in a table (Figure 2) that displays, for each signaled pathogen, the week in which the increase occurred (by week of sampling), the type of algorithm used, and the epidemiologic week in which the signal was generated.

Signals remain in this table for one epidemiologic week after they are signaled. For each signaled pathogen, a link can be made to a graph showing the observed and threshold for the previous 2 years. Historical signals by week of signaling are also listed on the site. Age and sex breakdown of all cases of a pathogen in the previous 4 weeks can be compared with that of all data, allowing an idea of which age or sex may be affected in an outbreak. Those with access to the site can also subscribe to automatically receive an email of a new signal.

## Signal Interpretation and Action

Signals that were produced during the previous 7 days are interpreted formally on a weekly basis in a meeting of members of RIVM and the National Co-ordination Centre for Communicable Disease Outbreak Management. Since 1999, this group has interpreted all signals of potential national importance, from informal and formal sources. In addition, the algorithm-generated signals are monitored on a daily basis. The accessibility of the site allows input from many other health professionals who can contact ISIS should they have some information to help interpret any signal.

Every week a meeting report is written and disseminated to all 46 regional health authorities as well as to the Ministry of Health and other interested parties. The investigation and control of outbreaks within one area is the legal responsibility of that area’s health authority. For outbreaks that span one or more health authorities, the RIVM coordinates and supports investigation, while RIVM and the National Co-ordination Centre for Communicable Disease Outbreak Management coordinate implementation of control measures.

**Figure 3:**
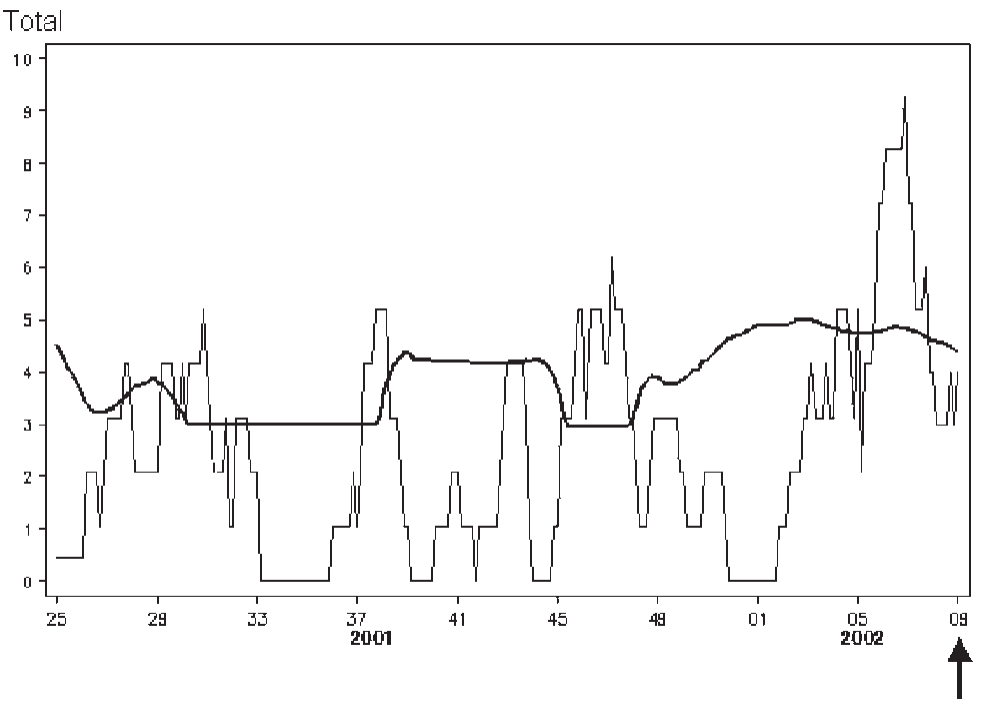
Graph showing sharp increase of weekly totals (week of sampling) of syphilis diagnoses (black line) exceeding the 99% threshold (red line). Arrow (week 9) marks when submitted laboratory reports resulted in signal generation and subsequent investigation.

The early-warning system was implemented in January 2002. In early March 2002, the system signaled an increase in diagnoses of syphilis. This increase was subsequently found to represent a sustained outbreak of syphilis that had begun the previous year in a large Dutch city. The outbreak was subsequently investigated, and prevention strategies were implemented (*21*) (Figures 2 and 3).

## Limitations

This system is designed to complement, not replace, any conventional methods of outbreak detection (e.g., clinician-based surveillance of notifiable diseases). Laboratory-based surveillance will be less timely and sensitive than conventional methods in detecting many local outbreaks of disease, particularly those clearly associated with a certain setting, and in detecting many widespread outbreaks of disease with unusual signs and symptoms (e.g., acute flaccid paralysis in a polio outbreak). Local outbreaks may also be more rapidly detected from local, not national, laboratory data. In addition, though expansion is planned, many laboratories are likely never to participate in ISIS, limiting the coverage of the electronic system.

Analysis of large amounts of laboratory data will likely signal many clusters of no significance, and the work generated in interpreting signals meaningfully may be overwhelming and so mask true signals. Thoroughly evaluating and adjusting parameters such as the minimum number required to trigger a signal may be required to prevent this but at a cost of losing sensitivity. Conversely, the ability to detect clusters of commonly reported pathogens that are not routinely subtyped (e.g., *Campylobacter*) will always be limited because the signal will be likely smaller than the variability of the large amount of data routinely submitted. One solution to this problem is to apply the algorithm to subsets of reduced amounts of data on common pathogens such as data collected by a group of regional laboratories.

## Future Work

### Evaluation of the System

The ISIS outbreak detection system needs to be evaluated to demonstrate a clear advantage over conventional means for detecting outbreaks of infection of all types of pathogens, not just salmonellae (for which the algorithm has already proved its usefulness). The sensitivity and timeliness of algorithms in other outbreak detection systems relative to a variety of standards such as formal records of investigated outbreaks or informal epidemiologic judgment, have been assessed retrospectively (*11*,*12*). However, no records of investigated outbreaks in the Netherlands exist, and the minutes from the signals meeting have only recently been put in a format that allows easy interpretation of signal outcome. In addition, retrospective analysis does not allow evaluation of the extra sensitivity nor of the specificity of an algorithm. This limitation exists because any signals from historical data produced by the algorithm, and not detected by other means, are classified as false positives, when many may have been genuine. Nonetheless, some idea of the value of the algorithm is given by the fact that since 1998, no national outbreak of *Salmonella* has been detected by means other than by the *Salmonella* outbreak detection system. Additionally, the feedback on the Internet and comments from the public health community are important factors that affect the sensitivity, specificity, and timeliness of the whole system since they will impact the eventual interpretation of a signal.

ISIS will, therefore, be evaluated prospectively at the weekly signal meeting, comparing signals detected by the algorithm to signals detected by other means. This comparison will allow assessment of the following: 1) how many signals detected by the algorithm are not of public health interest, as decided in the weekly meeting (a measure of specificity), and 2) the number of relevant signals detected by other means that should have been detected by the algorithm (a measure of relative sensitivity and timeliness). Assessing the number of outbreaks that the algorithm detects that would not have been detected otherwise will not be possible, since once a signal is detected by algorithm it can never be known with certainty that it would not have been detected later by other means. However, if the first detection of a signal is by algorithm, this will give some measure of timeliness of the system.

## Expansion

At present, 40 surveillance diagnoses in ISIS are available for use in the automated outbreak detection system. Much incoming data are as yet not formatted for daily signal generation and feedback as described. A priority, therefore, is to adapt the system to directly analyze raw data (those not processed as surveillance diagnoses) on the other 300 pathogens currently collected, and, in particular, to make the current *Salmonella* outbreak detection system part of the automated ISIS. By 2004, a total of 25 laboratories are scheduled to be connected, increasing the coverage of the system for all pathogens to at least 35% of the Dutch population. We also hope that regional health authorities will eventually have access to their own Web page, presenting the results of applying the algorithms to their data. This improvement would allow smaller regional outbreaks of common pathogens to be detected.

## Conclusion

We describe an automated outbreak detection system that uses laboratory data electronically collected in the Netherlands by ISIS. The system assesses data as soon as they are made available and disseminates the information by means of the Internet to all involved health professionals to help in the rapid interpretation and subsequent action to control any suspected outbreak. Much still needs to be done, and efforts are now concentrated on increasing the data available to ISIS, system evaluation, and subsequent modifications, with the aim of having a flexible, automated outbreak detection system for all laboratory-reported pathogens in the Netherlands by 2006.
